# On‐Chip Annealing Using Embedded Micro‐Heater for Highly Sensitive and Selective Gas Detection

**DOI:** 10.1002/advs.202401821

**Published:** 2024-05-13

**Authors:** Jinwoo Park, Hunhee Shin, Gyuweon Jung, Seongbin Hong, Min‐Kyu Park, Joon Hwang, Jong‐Ho Bae, Jae‐Joon Kim, Jong‐Ho Lee

**Affiliations:** ^1^ Department of Electrical and Computer Engineering and Inter‐university Semiconductor Research Center Seoul National University Seoul 08826 Republic of Korea; ^2^ School of Electrical Engineering Kookmin University Seoul 02707 Republic of Korea

**Keywords:** differential amplifier, FET‐type gas sensor, metal oxide, on‐chip annealing

## Abstract

The demand for gas sensing systems that enable fast and precise gas recognition is growing rapidly. However, substantial challenges arise from the complex fabrication process of sensor arrays, time‐consuming data transmission to an external processor, and high energy consumption in multi‐stage data processing. In this study, a gas sensing system using on‐chip annealing for fast and power‐efficient gas detection is proposed. By utilizing a micro‐heater embedded in the gas sensor, the sensing material of adjacent sensors in the same substrate can be easily varied without further fabrication steps. The response to oxidizing gas is constrained in metal oxide (MOX) sensing material with small grain sizes, as the depletion width of grain cannot extend beyond the grain size during the gas reaction. On the other hand, the response to reducing gases and humidity, which decrease the depletion width, is less affected by grain sizes. A readout circuit integrating a differential amplifier and dual FET‐type gas sensors effectively emphasizes the response to oxidizing gases by canceling the response to reducing gases and humidity. The selective on‐chip annealing method is applicable to various MOX sensing materials, demonstrating its potential for application in commercial fields due to its simplicity and expandability.

## Graphical abstract

By utilizing embedded micro‐heaters for post‐deposition annealing, metal oxide grain size and gas responses of adjacent sensors in the same substrate can be controlled without further fabrication steps. The proposed dual FET‐type gas sensors with on‐chip annealing can directly determine the gas concentration in a mixed gas environment, introducing a new approach for a real‐time energy‐efficient gas sensing system.

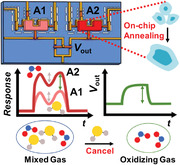

## 1 Introduction

Gas sensors have become vital tools in a wide range of applications, such as air quality monitoring,^[^ ^1^ ^]^ medical diagnosis,^[^ ^2^ ^]^ and food freshness determination.^[^ ^3^ ^]^ The growing demand for gas sensors has motivated extensive research on artificial olfactory systems (AOSs) capable of sensing and distinguishing multiple gases.^[^ ^4^, ^5^, ^6^ ^]^ Conventional AOSs employ gas sensor arrays integrated with several sensors to collect response patterns. The analog signals from sensor arrays are converted into digital signals through analog‐to‐digital converters. The converted signals are subsequently transferred to external microprocessors^[^ ^7^ ^]^ or servers^[^ ^8^ ^]^ and processed using pattern recognition methods^[^ ^9^ ^]^ or artificial neural networks (ANNs).^[^ ^10^ ^]^ This data transfer between the sensor array and the microprocessor involves challenges, particularly in energy consumption and latency. The on‐chip computation that integrates the gas sensing system and the interface circuit on the same substrate can be an attractive solution to reduce power consumption and signal delay effectively.^[^ ^11^, ^12^, ^13^ ^]^ However, most gas sensors are not compatible with the conventional complementary metal oxide semiconductor (CMOS) process, and there are not enough studies focused on integrating gas sensors with CMOS‐based interface circuits.

Manufacturing a large number of sensors with distinct gas sensing characteristics is also essential for AOSs. A number of sensing materials have been investigated for sensing diverse target gases. Among the various sensing materials, metal oxide (MOX) has emerged as a promising choice due to its high sensitivity and cost‐effectiveness. In particular, film‐type MOXs manufactured by diverse methods, including sol‐gel process,^[^ ^14^ ^]^ evaporation,^[^ ^15^ ^]^ and sputtering,^[^ ^16^ ^]^ are being studied extensively. However, the low selectivity of film‐type MOX gas sensors makes their application in gas mixtures challenging. Numerous efforts have been made to enhance selectivity and detection limits by manipulating sensing material characteristics. One potential solution is to utilize secondary materials such as metal dopants^[^ ^17^ ^]^ or functional polymers.^[^ ^18^, ^19^ ^]^ For example, SnO_2_ and WO_3_ film‐type gas sensor arrays doped with diverse metal dopants (Pd, Pt, Au, and Bi) successively detected volatile organic compounds and wine samples using ANNs.^[^ ^20^, ^21^ ^]^ Although introducing additional materials can increase the selectivity of specific gases, they still face issues related to uniformity and reliability, which are essential for commercialization.

A more straightforward approach to modifying gas sensing characteristics of MOX involves changing post‐deposition annealing (PDA) conditions. The properties of MOX are significantly influenced not only by the deposition method but also by the PDA conditions, including annealing time, temperature, and atmosphere. Korotcenkov, G. et al. analyzed changes in In_2_O_3_ film properties, including structure stability, morphology, and grain size, after PDA in the temperature range from 500 to 1100 °C.^[^ ^22^ ^]^ These property changes in MOX significantly impact the gas sensing performance of MOX. Conventional PDA processes employ external annealing equipment such as a hot plate. Therefore, it is difficult to adjust different PDA conditions for multiple sensors on a single chip because the sensors are exposed to the same temperature and atmosphere simultaneously.

**Figure 1:**
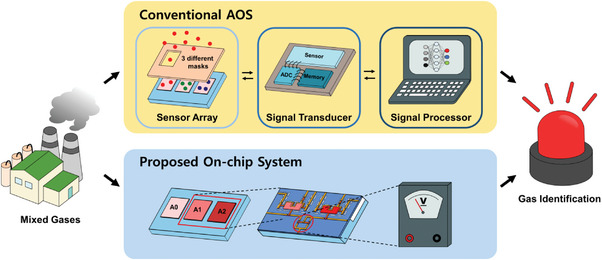
Schematic diagram of conventional AOS and proposed on‐chip gas sensing system. The proposed system eliminates the need for complex sensor fabrication steps and resolves power consumption and latency issues arising from data transmission and multi‐stage signal processing.

In this work, we present a novel approach to adjust gas sensitivity through the selective on‐chip annealing process (**Figure** **1**). With a differential amplifier readout circuit, CMOS‐compatible dual FET‐type gas sensors can distinguish and amplify the response of oxidizing gases in mixed gas environments. The FET‐type gas sensor has advantages such as compatibility with CMOS, small size, and high yield, making it suitable for application to edge devices.^[^ ^23^, ^24^, ^25^, ^26^ ^]^ The output voltage does not need further signal processing steps, addressing latency issues commonly encountered in conventional AOSs. The micro‐heaters embedded in FET‐type gas sensors operate for both the gas response and the on‐chip annealing process. In our on‐chip annealing process, the PDA condition can be easily varied for each sensor by adjusting the annealing voltage and time. The grain size growth with on‐chip annealing significantly enhances gas responses to oxidizing gases, while its influence on reducing gases and humidity is negligible. By employing grazing incidence X‐ray diffraction (GIXRD), scanning electron microscopy (SEM), and atomic force microscopy (AFM) analyses, we demonstrate the effects of on‐chip annealing on the sensing material and propose a mechanism underlying the change in gas sensing characteristics. The differential amplifier can emphasize the difference between responses of dual FET‐type gas sensors with different annealing conditions, deriving the accurate concentration of oxidizing gases unaffected by the presence of reducing gases and humidity. Circuit operation in a gas mixture environment is validated through circuit simulation based on actual measured gas sensing data.

## 2 Results and Discussion

### 2.1 Electrical Properties of FET‐Type Gas Sensor

**Figure 2:**
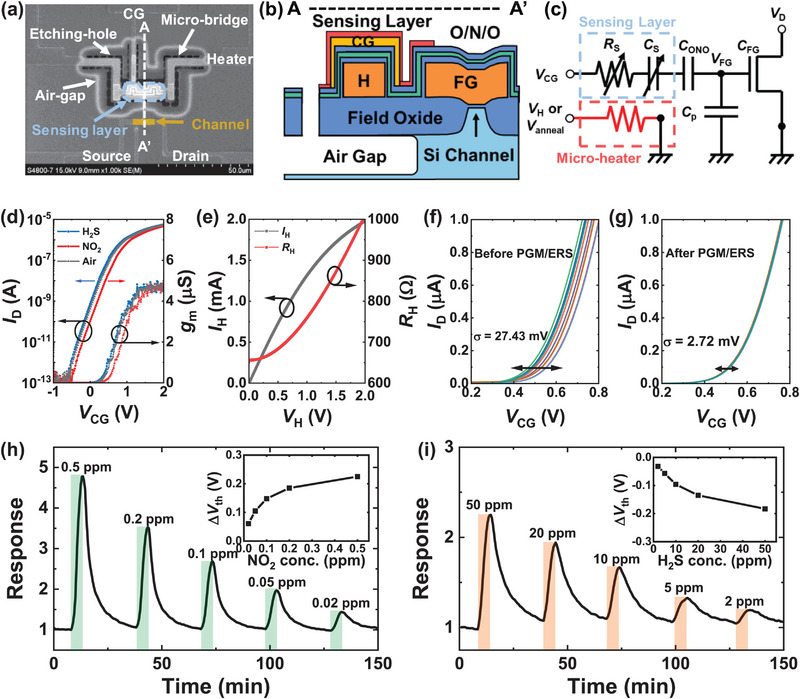
Characteristics of FET‐type gas sensor platform. a) Top SEM image of the fabricated gas sensor with an embedded micro‐heater. b) Cross‐sectional schematic view of the sensor cut along the dashed line (A‐A’). Here, H and FG represent the embedded micro‐heater and floating gate composed of heavily doped *n* ^+^, respectively. c) Simplified equivalent circuit diagram of the FET‐type gas sensor. d) *I*_D_–*V*_CG_ and *g*_m_ of the FET‐type gas sensor before and after exposure to 0.5 ppm NO_2_ and 50 ppm H_2_S gas. e) Voltage‐dependent current and resistance characteristics of the embedded micro‐heater. *I*_D_–*V*_CG_ curves of 10 FET‐type gas sensors f) before and g) after PGM/ERS operations to resolve *V*_th_ mismatch. Transient responses of the FET‐type gas sensor to h) NO_2_ and i) H_2_S gas at various gas concentrations. The sensor is operated at *V*_H_ of 1.0 V, and Δ*V*_th_ versus gas concentrations is presented in the inset.

**Figure** **2**a,b shows the top SEM image and cross‐sectional schematic view of the fabricated FET‐type gas sensor platform with an embedded micro‐heater (H) and In_2_O_3_ sensing layer. The horizontally formed control gate (CG) and floating gate (FG) are interdigitated to increase the coupling ratio between the CG and FG. The simplified equivalent circuit of the FET‐type gas sensor is illustrated in Figure 2c. *R*_S_ consists of the contact resistance with the CG and the bulk resistance of the sensing layer. The FG‐channel capacitance, the parasitic capacitance, the sensing layer capacitance, and the O/N/O passivation layer capacitance are denoted as *C*_FG_, *C*_P_, *C*_S_, and *C*_ONO_, respectively. The gas sensing mechanism of the FET‐type gas sensor is explained by the adsorption and desorption of gas molecules on the In_2_O_3_ sensing material, which is in contact with the CG and the passivation layer covering the FG.^[^ ^27^ ^]^ For example, oxidizing NO_2_ gas molecules take electrons from In_2_O_3_ film and are adsorbed as nitrite (NO_2_^−^) or nitrate (NO_3_^−^) ions depending on the operating temperature.^[^ ^28^ ^]^ The adsorbed ions act as excessive negative charges at the O/N/O interface near FG and shift the *I*_D_–*V*_CG_ curve of the sensor in a positive direction. In contrast, reducing H_2_S gas molecules induces positive charges, which shift the *I*_D_–*V*_CG_ curve in a negative direction. The *I*_D_–*V*_CG_ curves at *V*_D_ = 0.1 V and transconductance (*g*_m_)–*V*_CG_ curves before and after response to NO_2_ and H_2_S gas are plotted in Figure 2d. Note that the *I*_D_–*V*_CG_ curves show parallel shifts in the *x*‐direction by gas reactions while maintaining maximum *g*_m_ and subthreshold swing constant.

Figure 2e shows voltage‐dependent characteristics of the current and resistance of the embedded *n* ^+^‐doped poly‐silicon micro‐heater. As the applied voltage increases, the resistance of the heater (*R*_H_) increases due to the self‐heating of the micro‐heater. The air gap is formed under a poly‐silicon micro‐heater, ensuring excellent thermal insulation and power efficiency. Moreover, the FET transducer is separated from the micro‐heater. This separation allows the Si channel to remain at room temperature, which reduces baseline drift and noise during sensing operations.^[^ ^29^, ^30^ ^]^ The temperature of the micro‐heater as a function of power consumption and *V*_H_ is plotted in Figure S1 (Supporting Information). The temperature is estimated by the change in *R*_H_. The temperature estimation method has been compared to thermo‐reflectance microscopy and infrared micro‐thermography in previous works, and the accuracy of the method has been assured.^[^ ^23^, ^31^ ^]^ The micro‐heater operates reliably in *V*_H_ from 1.0 V (140 °C) to 1.7 V (320 °C) (Figure S2, Supporting Information), showing negligible degradation in *R*_H_. Owing to its stable operation even at temperatures higher than the typical gas response temperature, the micro‐heater can be used for a wide range of annealing conditions.

The *I*_D_–*V*_CG_ curves of 10 FET‐type gas sensors fabricated on the same wafer are plotted in Figure 2f. The *V*_th_ of sensors shows a narrow distribution (σ(*V*_th_) = 27.43 mV), ensuring a uniform and reliable sensor fabrication process. Figure 2g shows the *I*_D_–*V*_CG_ curves (σ(*V*_th_) = 2.72 mV) tuned by PGM/ERS operations. The *V*_th_ variation before and after PGM/ERS operations is depicted in Figure S3 (Supporting Information). The small mismatch in *V*_th_ can be further adjusted through program/erase (PGM/ERS) operations since the sensor platform has flash memory capability to store charge in the FG. When a PGM (ERS) pulse is applied to the CG while the body, source, and drain are grounded, electrons (holes) in the FET channel are injected into the FG via Fowler‐Nordheim tunneling, resulting in a shift of *I*_D_–*V*_CG_ curve in a positive (negative) direction. The sensor shows good retention characteristics in both programmed and erased states.^[^ ^32^ ^]^

The gas responses for oxidizing and reducing gases are defined as the ratios *I*_air_/*I*_gas_ and *I*_gas_/*I*_air_, respectively. *I*_air_ and *I*_gas_ represent the drain current of the gas sensor in the dry air (ambient atmosphere) and after the injection of target gas for 10 minutes. The response is obtained under the condition that *V*_CG_ equals *V*_th_ at *V*_D_ = 0.1 V to maximize the signal‐to‐noise ratio unless specifically mentioned otherwise.^[^ ^33^ ^]^ *V*_th_ is extracted using the constant‐current method, and the *V*_CG_ at which the drain current is equal to 100 nA is defined as *V*_th_. Figure 2h,i shows the transient response of the FET‐type gas sensor to NO_2_ gas and H_2_S gas, respectively, with varying concentrations of target gases at *V*_H_ of 1.0 V. Δ*V*_th_ versus gas concentration is presented in the inset. The response to 0.5 ppm NO_2_ and 50 ppm H_2_S gas as a function of *V*_H_ is illustrated in Figure S4 (Supporting Information). The optimal *V*_H_ of NO_2_ gas detection is 1.1 V, while the response to H_2_S gas increases with rising temperature until 1.2 V. The limit of detection (LOD) is calculated to be 0.79 ppb, and the detailed calculation process is shown in Figure S5 (Supporting Information).^[^ ^34^ ^]^ **Table** **1** summarizes the features of reported ppb‐level NO_2_ gas sensors with different transducer types.^[^ ^35^, ^36^, ^37^, ^38^, ^39^ ^]^ Our FET‐type gas sensor shows the best power efficiency (140 °C at 1.18 mW) and sub‐ppb LOD.

**Table 1:** Comparison between reports on ppb‐level NO_2_ gas sensors and present work.

Sensor type	Sensing material	Response [@100 ppb]	Power [operating temperature]	Response time	LOD	Ref.
Resistor	Pt/SnO_2_ Nanotube array	1.3	40 mW (200 °C)	×10^2^ s	0.1 ppb	^[^ ^35^ ^]^
Resistor	Au/Co_3_O_4_ Nanoparticles	1.34	11.2 mW (136 °C)	84 s	10 ppb	^[^ ^36^ ^]^
Resistor	WO_3_ Thin film	1.6	6.6 mW (150 °C)	×10^2^ s	16 ppb	^[^ ^37^ ^]^
FET	MoS_2_ Monolayer	1.5	RT	×10^2^ s	10 ppb	^[^ ^38^ ^]^
FET	Single‐wall Carbon nanotube	2	RT	×10^2^ s	86 ppb	^[^ ^39^ ^]^
FET	In_2_O_3_ Thin film	2.68	1.18 mW (140 °C)	×10^2^ s	0.79 ppb	This work

### 2.2 Material Characterization of In_2_O_3_ Sensing Layer

**Figure 3:**
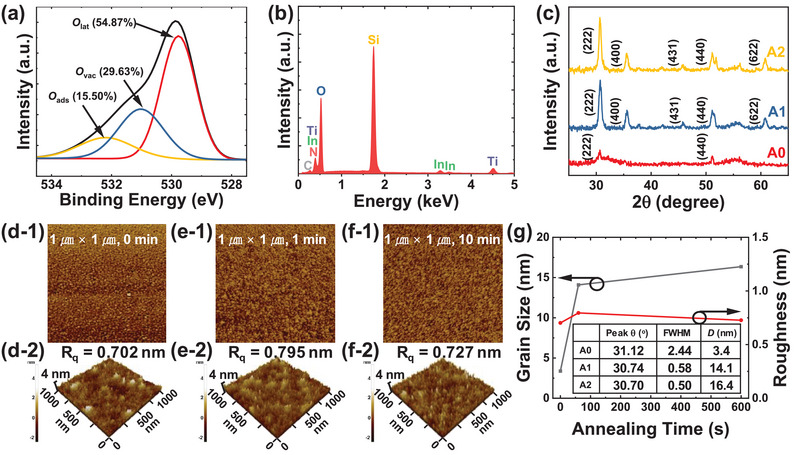
Material characterization of In_2_O_3_ thin film. a) High‐resolution O 1s XPS spectrum and b) EDS analysis of as‐deposited In_2_O_3_ film. c) GIXRD patterns of A0, A1, and A2 films. A0, A1, and A2 films are annealed for 0, 1, and 10 min at 270 °C, respectively. Topographic AFM images of d‐1) A0, e‐1) A1, and f‐1) A2 films. 3D surface morphologies of (d‐2) A0, (e‐2) A1, and (f‐2) A2 films. All the images are obtained for 1 µm × 1 µm surface area. g) Surface roughness from AFM analysis and grain size calculated from GIXRD patterns as a function of annealing time.

To examine the characteristics of the as‐deposited sensing material, quantitative analyses are performed using X‐ray photoelectron spectroscopy (XPS) and energy dispersive spectroscopy (EDS). Figure S6 (Supporting Information) shows a wide‐scan XPS spectrum of In_2_O_3_ thin film. The oxygen‐to‐indium ratio of the film surface is determined to be 1.48, which is close to the stoichiometric value of In_2_O_3_. The high‐resolution XPS spectrum of O 1s is deconvoluted into three peaks with binding energies of 529.77, 531.02, and 532.19 eV (**Figure** **3**a). Three peaks correspond to lattice oxygen (*O*_lat_), oxygen adjacent to oxygen vacancy (*O*_vac_), and surface oxygen species (*O*_ads_), respectively.^[^ ^40^, ^41^ ^]^ In EDS analysis, the presence of indium and oxygen elements is confirmed, and scarce impurity elements are observed (Figure 3b).

Various analytical methods are conducted to verify the impact of annealing on the sensing materials. The In_2_O_3_ films with different annealing times (0, 1, and 10 min) are defined as A0, A1, and A2 films, respectively. The films are deposited with 15 nm thickness on silicon oxide substrates and annealed using an external chuck at 270 °C. GIXRD is used to analyze the grain size and crystallinity of the film (Figure 3c). The as‐deposited In_2_O_3_ film (A0) shows an amorphous phase. After annealing, characteristic peaks (222), (400), (431), (440), and (622) of polycrystalline cubic In_2_O_3_ (JCPDS 06–0416) are observed. The peak corresponding to (222) is the strongest, indicating a preference for the (222) crystal plane direction in annealed In_2_O_3_ films. 1 µm × 1 µm topographic AFM images and 3D surface morphologies of A0, A1, and A2 films are illustrated in Figure 3d–f. All films exhibit uniform and granular structure, while the grain size increases with extended annealing times. On the other hand, the root‐mean‐square roughness remains nearly constant within the range of 0.7 to 0.8 nm. The average grain size (*D*) is estimated from GIXRD patterns by following Scherrer equation:^[^ ^42^ ^]^

(1) $$D=\frac{K\lambda }{\beta \cos\theta }$$

where *K*, *λ*, *β*, and *θ* stand for the shape factor, the wavelength of the X‐ray, the full width at half the maximum (FWHM) intensity, and the diffraction angle. Assuming spherical particles, *K* is set to 0.9. The estimated *D*s of A0, A1, and A2 films using the Scherrer equation are 3.4, 14.1, and 16.4 nm, respectively, as depicted in Figure 3g. AFM and GIXRD analyses demonstrate the In_2_O_3_ grain growth with the rise in annealing time.

### 2.3 On‐Chip Annealing Effect on Gas Response

**Figure 4:**
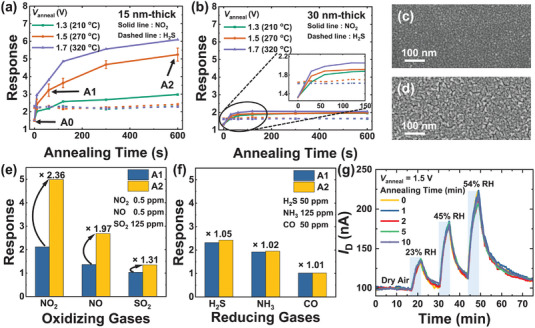
On‐chip annealing effect on FET‐type gas sensor response. Response to 0.5 ppm NO_2_ and 50 ppm H_2_S gas versus on‐chip annealing time of a) 15 nm and b) 30 nm thick FET‐type gas sensors as a parameter of *V*_anneal_. All sensors are operated at a *V*_H_ of 1.0 V (140 °C) during gas reaction. The 15 nm thick gas sensors annealed at *V*_anneal_ of 1.5 V with different annealing times (0, 1, and 10 min) are defined as A0, A1, and A2 sensors, respectively. Surface SEM images of c) 15 nm and d) 30 nm thick In_2_O_3_ as‐deposited films. Response of A1 and A2 sensors for various e) oxidizing (NO_2_, NO, and SO_2_) and f) reducing gases (H_2_S, NH_3_, and CO). g) Transient humidity responses as a parameter of annealing time (*V*_anneal_ = 1.5 V). Humid air with relative humidity (RH) from 23% to 54% is injected and recovered in dry air.

Using an embedded micro‐heater enables the application of diverse annealing conditions to multiple sensors on the same chip. **Figure** **4**a,b shows the gas response to NO_2_ and H_2_S gas versus annealing time as a parameter of annealing voltage (*V*_anneal_) applied to the micro‐heater with different In_2_O_3_ thicknesses. To verify the effect of film thickness on on‐chip annealing, two sensors with different In_2_O_3_ thicknesses (15 and 30 nm) are fabricated using identical radio frequency (RF) sputtering power and deposition rates. The film thickness is confirmed by the surface profiler, and surface SEM images of the as‐deposited films are shown in Figure 4c,d. The measured grain size is about five times larger in 30 nm film than in 15 nm film. It is well‐known that grain size is largely affected by the thickness of the sputtered film.^[^ ^43^, ^44^, ^45^, ^46^ ^]^ The longer deposition times lead to increased agglomerations, resulting in larger grain sizes. Interestingly, the response to NO_2_ gas of the sensor with 15 nm thick In_2_O_3_ film increases with extended annealing times, while the sensor with 30 nm thick In_2_O_3_ film shows a lower gas response increase by on‐chip annealing. All sensors are operated at *V*_H_ of 1.0 V (140 °C) during the gas reaction. As *V*_anneal_ increases, the response increases faster with annealing time and is saturated in higher response. Note that the response to H_2_S gas is not increased by the annealing process in sensors with both thicknesses. The on‐chip annealed thin film gas sensors with *V*_anneal_ of 1.5 V (270 °C) with different annealing times (0, 1, and 10 min) correspond to A0, A1, and A2 sensors, respectively. The average and standard deviation of the responses of sensors annealed with *V*_anneal_ of 1.5 V are calculated using the results from five sensors on one chip (Figure 4a). Response to NO_2_ gas increases by 1.60 times in the A1 sensor and 3.47 times in the A2 sensor compared to the response of the A0 sensor. Figure S7 (Supporting Information) depicts the repeated transient change in the *I*_D_ of three sensors. The process of injecting 0.5 ppm of NO_2_ gas for 10 min and recovering in dry air is repeated seven times. The response of the A0 sensor gradually increases while the A1 and A2 sensors show identical cyclic response characteristics. When annealed for longer than 1 min, the increased response is maintained stably without further increase in repeated gas reactions. While the A2 sensor shows a more dramatic increase in response when compared to the A0 sensor, it is appropriate to compare it to the A1 sensor for reliable and repeatable gas response results.

The responses to various gases (0.5 ppm NO_2_, 0.5 ppm NO, 125 ppm SO_2_, 50 ppm H_2_S, 125 ppm NH_3_, and 50 ppm CO) of A1 and A2 sensors are shown in Figure 4e,f. The response to NO_2_ gas is increased by 2.36 times, and the enhancement in response is not limited to NO_2_ gas. The response to oxidizing gases (NO_2_, NO, and SO_2_) increases significantly with on‐chip annealing, whereas the response to reducing gases (H_2_S, NH_3_, and CO) shows negligible change. Moreover, the humidity response of the sensor with different annealing times is examined in Figure 4g. Dry air and humid air (relative humidity (RH) from 23% to 54%) are alternatively injected. The sensors show identical responses to humidity regardless of the annealing time, which is similar to the behavior observed with reducing gases.

**Figure 5:**
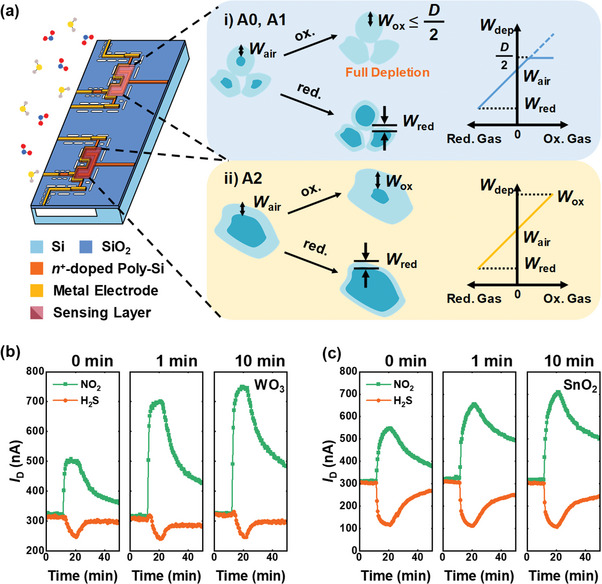
a) Schematic diagram of depletion width (*W*_dep_) limitation effect. The response to oxidizing gas is different depending on the grain size (*D*), while the response to reducing gas is less affected. The mechanism can be explained as follows: i) In MOX with small grains (A0 and A1), *W*_ox_ (depletion width due to oxidizing gas reaction) is limited by grain size as it reaches full depletion quickly. ii) When grain size is large enough (A2), the limitation on *W*_ox_ caused by grain size is eliminated, supplying more electrons to oxidizing gases. The increase in grain size has less effect on the response to reducing gas as *W*_red_ (depletion width due to reducing gas reaction) is not constrained. Transient responses to 0.5 ppm NO_2_ and 50 ppm H_2_S gas of b) WO_3_ and c) SnO_2_ sensors with different on‐chip annealing times. On‐chip annealing is performed at *V*_H_ of 1.5 V for 1 and 10 min.

In our study, the grain size of In_2_O_3_ is significantly smaller than the grain size of regular nanocrystals or thick films.^[^ ^47^, ^48^ ^]^ A number of studies have reported that reactivity to oxidizing gases increases as the grain size grows under 20–30 nm.^[^ ^49^, ^50^, ^51^ ^]^ The enhancement in response to oxidizing gases can be explained through the depletion width limitation effect in small grains (**Figure** **5**a). The depletion widths at the grains of the MOX film in dry air, oxidizing gas, and reducing gas are denoted as *W*_air_, *W*_ox_, and *W*_red_, respectively. In a dry air atmosphere, the grain of MOX is depleted to *W*_air_ due to adsorbed oxygen species. In MOX with small grains, such as A0 and A1 films, the grain quickly reaches full depletion when oxidizing gases are injected, and additional gas molecules cannot be adsorbed. With an increase in grain size, the MOX can supply more electrons to oxidizing gases before reaching full depletion, thereby enhancing the initially limited gas response. When grain size is large enough, the limitation on *W*_ox_ caused by grain size is eliminated. On the other hand, reducing gases donate electrons to MOX and decrease *W*_air_ to *W*_red_. As *W*_red_ is not constrained by the grain size, responses to reducing gases are independent of the annealing time. Therefore, the on‐chip annealing effect is maximized in thin films with small grains, which show very limited response in their as‐deposited state.

As a proof of concept, FET‐type gas sensors with different MOX sensing materials are also fabricated. 15 nm thick SnO_2_ and WO_3_ films are deposited using the RF magnetron sputtering, and the gas response is observed. Figure 5b,c depicts the transient *I*_D_ when exposed to 0.5 ppm NO_2_ and 50 ppm H_2_S gas of sensors with different annealing times. On‐chip annealing (*V*_anneal_ = 1.5 V) is conducted for 1 and 10 min. When sensors are on‐chip annealed for 10 min, the response to NO_2_ gas increases by 1.40 times in WO_3_ and 1.29 times in SnO_2_ compared to the response of as‐deposited sensors. On the other hand, the response to H_2_S gas remains almost constant, increasing by 1.01 times in WO_3_ and 1.05 times in SnO_2_, after 10 min of on‐chip annealing. As expected, the response to NO_2_ increases while the response to H_2_S is maintained. Surface SEM images of as‐deposited and annealed SnO_2_ films are shown in Figure S8 (Supporting Information). This suggests that on‐chip annealing is not limited to specific sensing material and can be widely applicable to diverse thin film MOXs with small grains.

### 2.4 Dual FET‐Type Gas Sensor with Differential Amplifier

**Figure 6:**
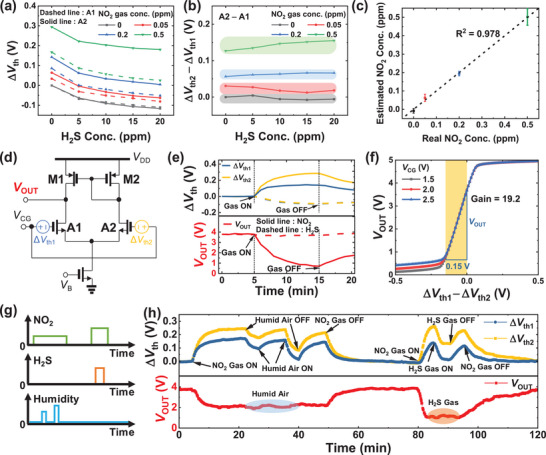
Dual FET‐type gas sensors using on‐chip annealing. a) ∆*V*_th_ of A1 and A2 sensors for mixtures of NO_2_ and H_2_S gas with various concentrations. b) Difference of ∆*V*_th_ of A1 and A2 sensors for gas mixtures. c) Estimated NO_2_ concentrations in various gas mixtures based on Langmuir adsorption isotherm fitted curve. d) Proposed circuit diagram of dual FET‐type gas sensors with differential amplifier. Δ*V*_th1_ and Δ*V*_th2_ due to gas response are reflected using the voltage sources connected between *V*_CG_ and gate of A1 and A2 sensors, respectively. e) Measured Δ*V*_th_ of A1 and A2 sensors exposed to 0.5 ppm NO_2_ and 50 ppm H_2_S gas and simulated *V*_OUT_ with SmartSPICE. f) Output characteristic of the proposed circuit as a parameter of *V*_CG_. The colored region shows the measured difference of ∆*V*_th_. g) Schematic diagrams of time‐dependent injection of NO_2_, H_2_S gas, and humid air. h) Measured Δ*V*_th_ of A1 and A2 sensors and simulated *V*_OUT_ in response to dynamic gas mixture injection. The *V*_OUT_ only reflects the NO_2_ gas concentration, acquiring resistance to H_2_S and humidity disturbance.

The enhanced response to oxidizing gases can be effectively applied in a mixed gas environment. **Figure** **6**a shows Δ*V*_th_s of A1 and A2 sensors when exposed to mixtures of NO_2_ and H_2_S gas in various concentrations. Several combinations of gases show the same Δ*V*_th_, which complicates the determination of specific gas concentrations. Although the A2 sensor shows increased response to NO_2_ gas, the gas response still shows a strong dependence on H_2_S gas concentration. This confusion in gas identification can be solved by utilizing the difference in responses of the two sensors using selective on‐chip annealing. Figure 6b shows the difference of Δ*V*_th_ of A1 and A2 sensors exposed to gas mixtures. The difference of Δ*V*_th_ is nearly constant for a given NO_2_ gas concentration, even if the H_2_S gas concentration varies from 0 to 20 ppm. As both sensors exhibit almost identical H_2_S gas response, the difference of Δ*V*_th_ is solely influenced by the NO_2_ gas concentration. Therefore, accurate identification of NO_2_ gas concentration is possible even in the presence of other reducing gases. Figure 6c depicts the NO_2_ concentration estimated using a fitted Langmuir equation. The difference of Δ*V*_th_ versus NO_2_ gas concentration curve in the absence of H_2_S gas is fitted based on the Langmuir adsorption isotherm (*R* ^2^ > 0.99) (Figure S9, Supporting Information).^[^ ^52^ ^]^ By utilizing dual FET‐type gas sensors with different on‐chip annealing conditions, the concentration of specific gas can be determined in gas mixtures.

A circuit diagram designed to detect the difference in response of dual FET‐type gas sensors (A1 and A2) is illustrated in Figure 6d. As each micro‐heater of dual FET‐type gas sensors can operate independently, on‐chip annealing conditions of dual sensors can be varied without any further fabrication process. The device characteristics (*V*_th_ and *g*_m_) of the dual sensors before gas response are identical. Note that *V*_th_ calibration is possible using PGM/ERS operation if the sensors exhibit *V*_th_ mismatch. Δ*V*_th_ due to gas response is simulated with the voltage source connected between *V*_CG_ and the gate of sensors. Δ*V*_th_ by gas response is transmitted to single‐ended output without loss of gain because the dual sensors are connected to the current mirror. The proposed circuit is simulated using SmartSPICE (Silvaco, Inc.) based on the measured transient gas response data. The MOSFET model parameters are calibrated with measured output characteristics of fabricated MOSFETs and gas sensors. Each device is designed to operate in the saturation region, and detailed device parameters and bias voltage conditions of the initial state are shown in Figure S10 (Supporting Information). Measured transient Δ*V*_th_ of A1 and A2 sensors when exposed to NO_2_ and H_2_S gas, along with the *V*_OUT_ curves, are presented in Figure 6e. Since the variations in *V*_th_ of A1 and A2 sensors are similar in the H_2_S gas response, *V*_OUT_ remains relatively constant during the H_2_S gas response and recovery. On the other hand, the variation in *V*_th_ of A2 is larger than that of the A1 sensor in the NO_2_ gas response, and *V*_OUT_ decreases from 3.7 to 0.7 V. Figure 6f shows *V*_OUT_ versus Δ*V*_th1_ – Δ*V*_th2_ of the proposed circuit as a parameter of *V*_CG_. The measured difference in the Δ*V*_th_ of the dual sensors when exposed to 0.5 ppm NO_2_ gas for 10 min is 0.15 V. In this region, the device operates within the saturation region and amplifies the signal properly. The proposed circuit shows a differential mode voltage gain of 19.2, amplifying Δ*V*_OUT_ to 2.88 V. When *V*_CG_ increases from 1.5 to 2.5 V, the variation in *V*_out_ is 0.026 V, and the common‐mode rejection ratio is calculated as 57.2 dB.

Figure 6g shows an example of gas mixture exposure with diverse combinations of gases (NO_2_ and H_2_S) and humid air. When additional H_2_S gas and humid air are injected during the NO_2_ gas response in a single sensor, the *V*_th_ experiences significant disturbances, as depicted in Figure 6h. Distinguishing whether NO_2_ gas alone exists in low concentration or coexists with reducing gases is challenging based solely on the single sensor. However, the dual FET‐type gas sensors with the differential amplifier can be effectively utilized in such circumstances. The *V*_OUT_ of the circuit remains relatively stable, changing only in response to the NO_2_ gas. This stability allows the accurate real‐time determination of the specific NO_2_ gas concentration within a mixture of NO_2_ and H_2_S gas (or humidity).

## 3 Conclusion

On‐chip annealing using embedded micro‐heaters in FET‐type gas sensors was proposed to distinguish oxidizing gas concentration in gas mixtures. The annealing condition of a single sensor can be selectively adjusted without additional fabrication steps. When on‐chip annealing was performed at 1.5 V for 10 min, the response to NO_2_ gas increased by 3.47 times, while the response to H_2_S gas was almost unchanged. Moreover, it showed improvement in responses to other oxidizing gases (NO and SO_2_) among diverse gases. This phenomenon can be explained by the full depletion of small grain sizes of thin films, which limited the adsorption of oxidizing gas molecules. Supporting the proposed mechanism, the growth of grain size in In_2_O_3_ thin films after annealing and varying film thickness was confirmed with SEM, AFM, and GIXRD analysis.

A simple differential amplifier circuit combined with dual FET‐type sensors was suggested and simulated using the circuit simulator SPICE. Based on the measured output and gas response characteristics of the FET‐type sensor, simulated results confirmed that Δ*V*_out_ accurately amplified the difference in gas reactivity. The proposed circuit enabled a precise determination of oxidizing gas concentration in mixed gas environments. Moreover, the expandability of the selective on‐chip annealing method on various sensing materials (SnO_2_ and WO_3_) was confirmed. We believe that the on‐chip annealing method will become an essential tool for modifying gas sensing characteristics, not only in terms of gas response but also in gas selectivity.

## 4 Experimental Section

### Gas Sensor Fabrication

Schematic cross‐sectional views of FET‐type gas sensor fabrication processes are illustrated in Figure S11 (Supporting Information). The fabrication began with a *p*‐type 6‐inch single‐crystalline Si wafer (1–10 Ω cm) with a (100) orientation. A 10 nm thick layer of SiO_2_ and a 150 nm thick Si_3_N_4_ layer were formed sequentially and patterned to define the active regions (Figure S11a, Supporting Information). The active regions were isolated by thermally grown 550 nm thick local oxidation of silicon. The Si_3_N_4_ and the SiO_2_ layers were removed sequentially. The sacrificial oxide layer was grown, and then ion implantation was performed to adjust *V*_th_. After the sacrificial oxide layer removal, a 10 nm thick gate oxide was grown by a dry oxidation process (Figure S11b, Supporting Information). A 350 nm thick in situ *n* ^+^‐doped poly‐silicon was deposited and patterned to form the FG and the micro‐heater, followed by the source/drain (S/D) implantation (Figure S11c, Supporting Information). Then, the SiO_2_/Si_3_N_4_/SiO_2_ (O/N/O, 10/20/10 nm) passivation layer was formed to protect the devices from the external environment (Figure S11d, Supporting Information). Heat treatment was performed to activate the implanted S/D impurities. After defining the contact holes, Ti/TiN/Al/TiN (20/30/100/20 nm) layers were deposited by RF magnetron sputtering and patterned using a lift‐off process to form the electrodes for the CG, S/D, and the micro‐heater (Figure S11e, Supporting Information). Etching holes were patterned by anisotropic etching of the field oxide. Si substrate was isotropically etched using a reactive ion etching process for the air‐gap formation under the heater (Figure S11f, Supporting Information). Finally, In_2_O_3_ sensing material was deposited using the RF magnetron sputtering method and patterned using a lift‐off process (Figure S11g, Supporting Information).

### Gas Sensing Measurement

The electric characteristics and gas sensing characteristics of the FET‐type gas sensor were measured using a semiconductor parameter analyzer (B1500A, Agilent). Figure S12 (Supporting Information) shows the experimental setup for gas sensing measurements. The target gases (NO_2_, H_2_S, NO, SO_2_, NH_3_, and CO) were diluted with dry air having an RH of 3.5% at 20 °C in the mixing chamber and introduced into the main chamber at a flow rate of 200 standard cubic centimeters per minute. For the humidity response measurement, dry air was bubbled through deionized water, and the mixing ratio of humid air and dry air was adjusted accordingly. The RH was confirmed through a commercial humidity sensor. Mass flow controllers (MFCs) regulate the gas flow rate appropriately, ensuring a controlled injection into the main chamber.

### In_2_O_3_ Characterization

Sensing material characteristics were analyzed using XPS (AXIS‐HSi, KRATOS) and EDS (AURIGA, Carl Zeiss). A conventional X‐ray source that emits Al *K*α radiation was used for XPS analysis. Surface images and crystallinity were obtained using SEM (SIGMA, Carl Zeiss) and GIXRD (Xpert Pro, PANalytical). The surface morphology of the film was examined using AFM (NX‐10, Park Systems) in tapping mode with a silicon cantilever.

## Conflict of Interest

The authors declare no conflict of interest.

## Supporting information

Supporting Information

## Data Availability

The data that support the findings of this study are available from the corresponding author upon reasonable request.
