# Supplementary material for: On‐Chip Annealing Using Embedded Micro‐Heater for Highly Sensitive and Selective Gas Detection
Source: Adv Sci (Weinh). 2024 May 13;11(28):2401821. doi: 10.1002/advs.202401821 (PMC11267278; doi:10.1002/advs.202401821)
Supplement: Supplementary file 1 — Supporting Information [file ADVS-11-2401821-s001.docx]

Supporting Information

On-chip annealing using embedded micro-heater for highly sensitive and selective gas detection

Jinwoo Park, Hunhee Shin, Gyuweon Jung, Seongbin Hong, Min-Kyu Park, Joon Hwang, Jong-Ho Bae, Jae-Joon Kim, and Jong-Ho Lee*


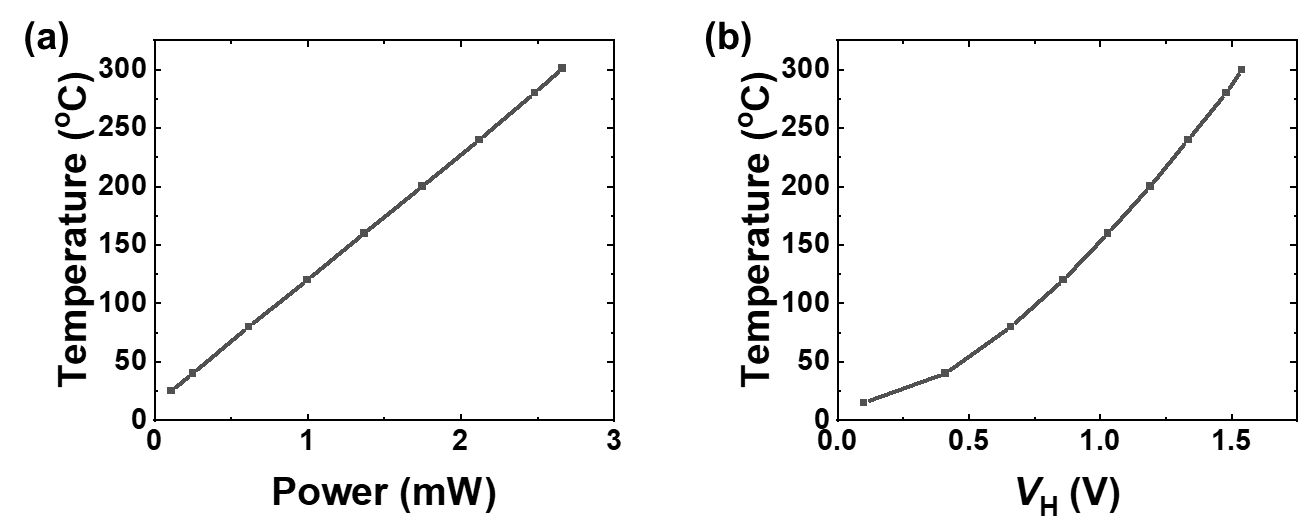


**Figure S1.** (a) Micro-heater temperature versus power consumption extracted through calculation from measurement results. (b) Micro-heater temperature versus V_H_.


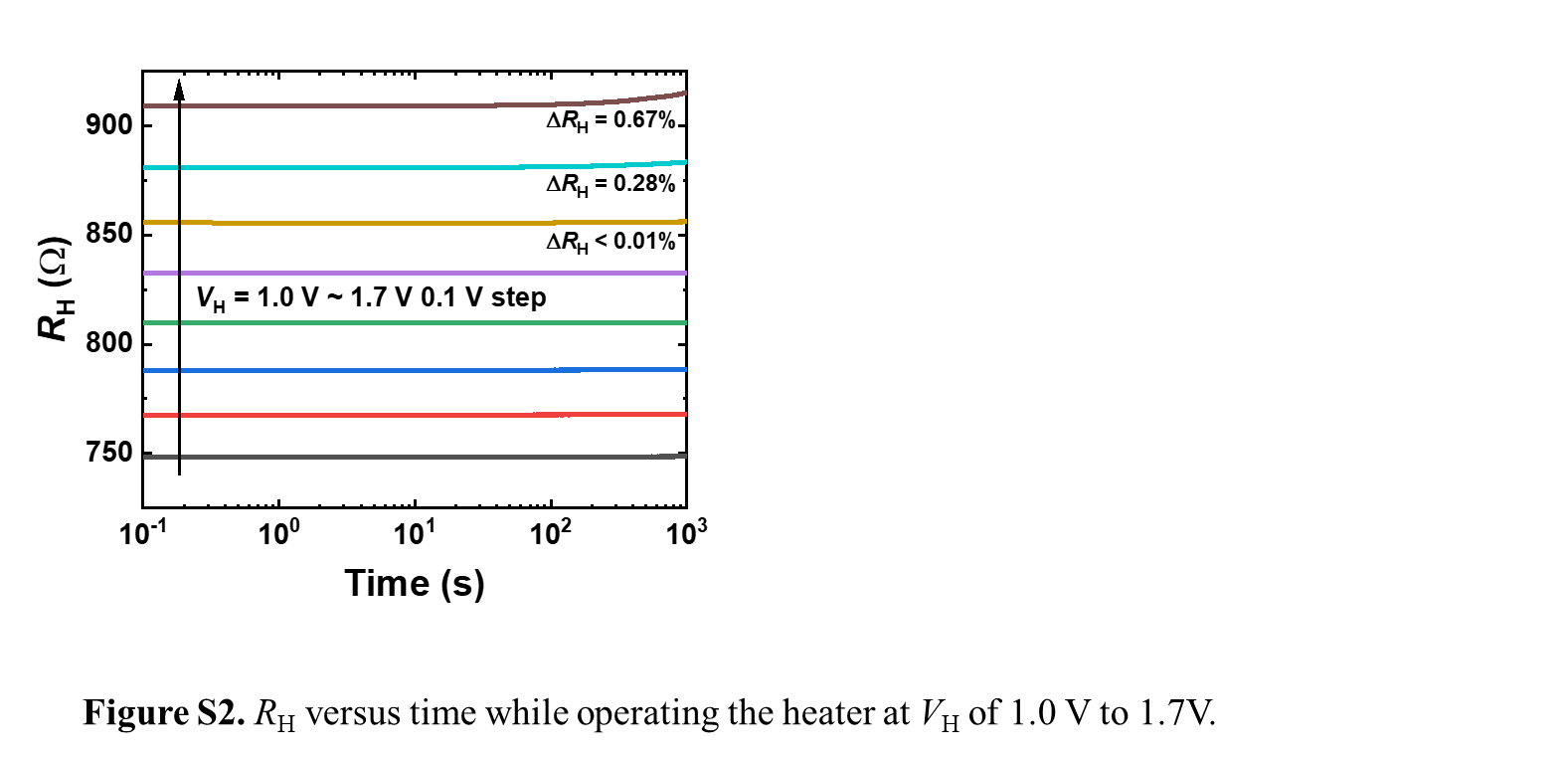


**Figure S2.** R_H_ versus time while operating the heater at V_H_ from 1.0 V to 1.7 V.

**
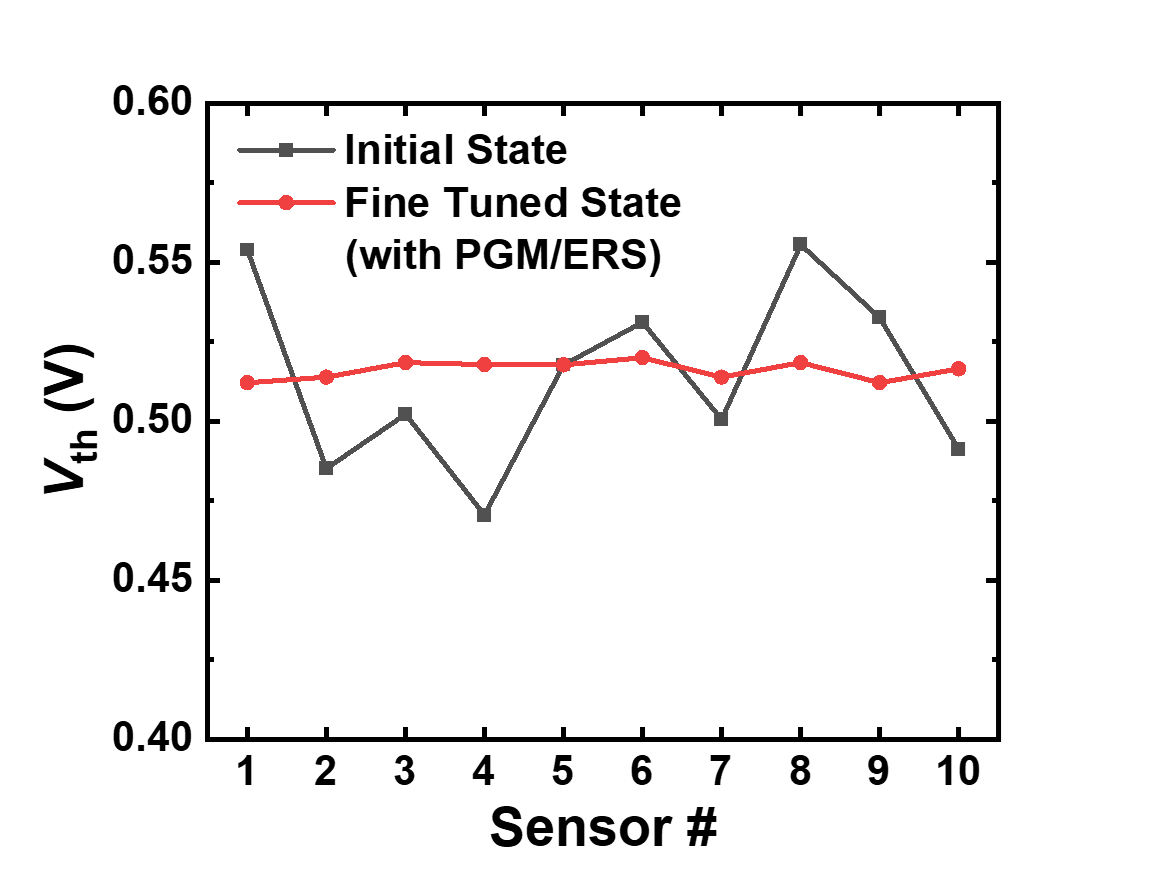
**

**Figure S3.** V_th_ variation of fabricated 10 gas sensors before and after PGM/ERS operations.

**Figure S4.** Response to 500 ppb NO_2_ and 50 ppm H_2_S gases obtained as a function of V_H_. The calculated temperature using the change in R_H_ of the micro-heater is shown on the upper x-axis.


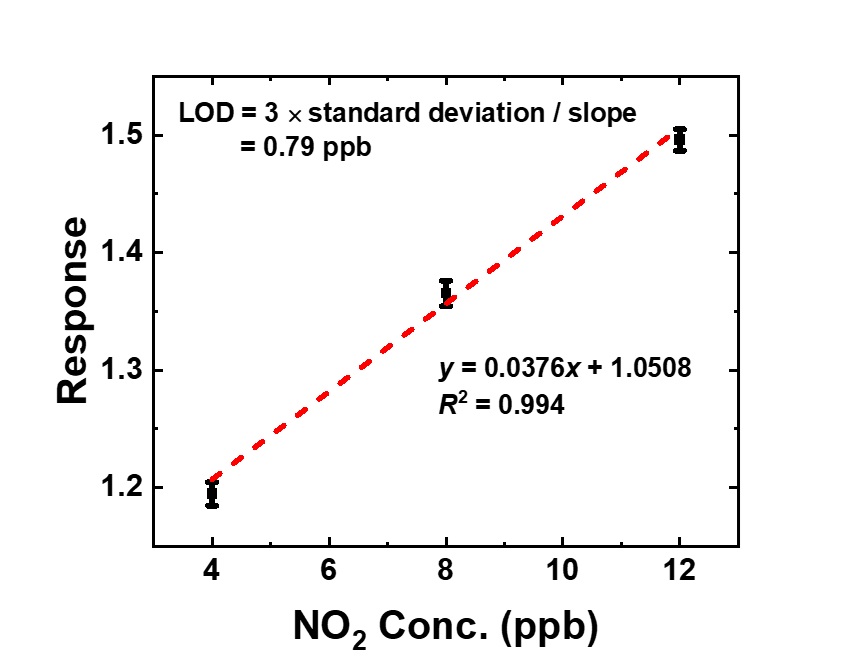


**Figure S5.** Gas response in low-concentration NO_2_ and theoretical LOD calculation.


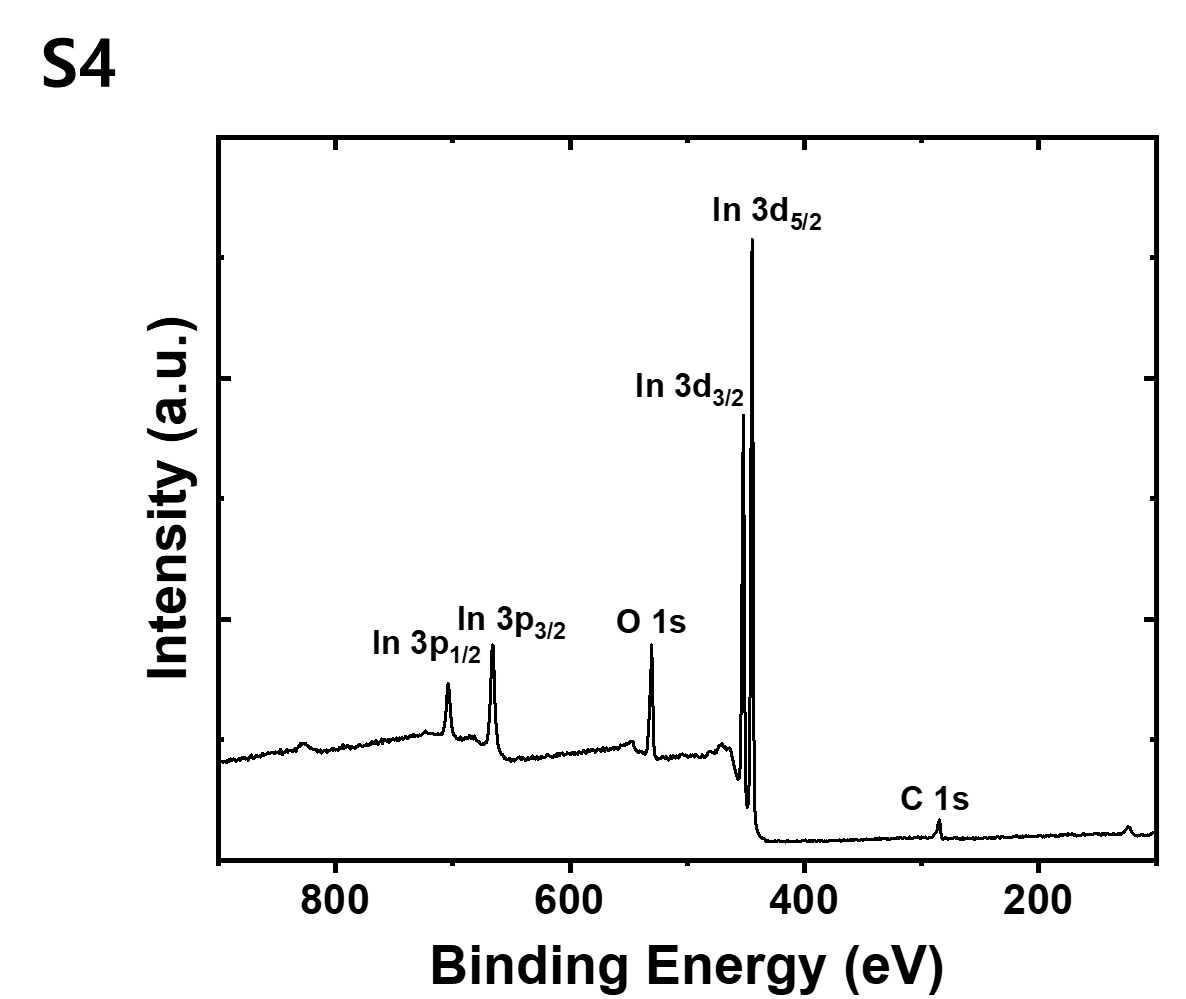


**Figure S6.** Wide-scan XPS spectrum of In_2_O_3_ film.

**Figure S7.** Repeated transient ∆I_D_ response to 500 ppb NO_2_ of A0, A1, and A2 sensors.


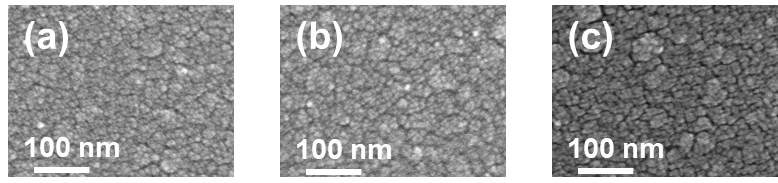


**Figure S8.** Surface SEM images of (a) as-deposited and annealed SnO_2_ films at 270 °C for (b) 1 minute and (c) 10 minutes.

**Figure S9.** Difference of ∆V_th_ of A1 and A2 sensors versus NO_2_ concentration. The curve is fitted based on the Langmuir adsorption isotherm (R^2^ > 0.99).


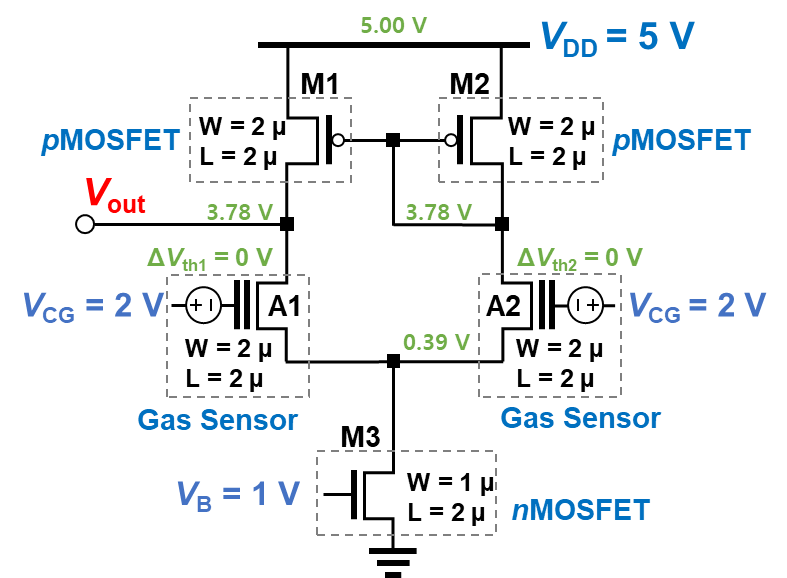


**Figure S10.** Device parameters and bias voltage conditions at the initial state used in circuit simulation.


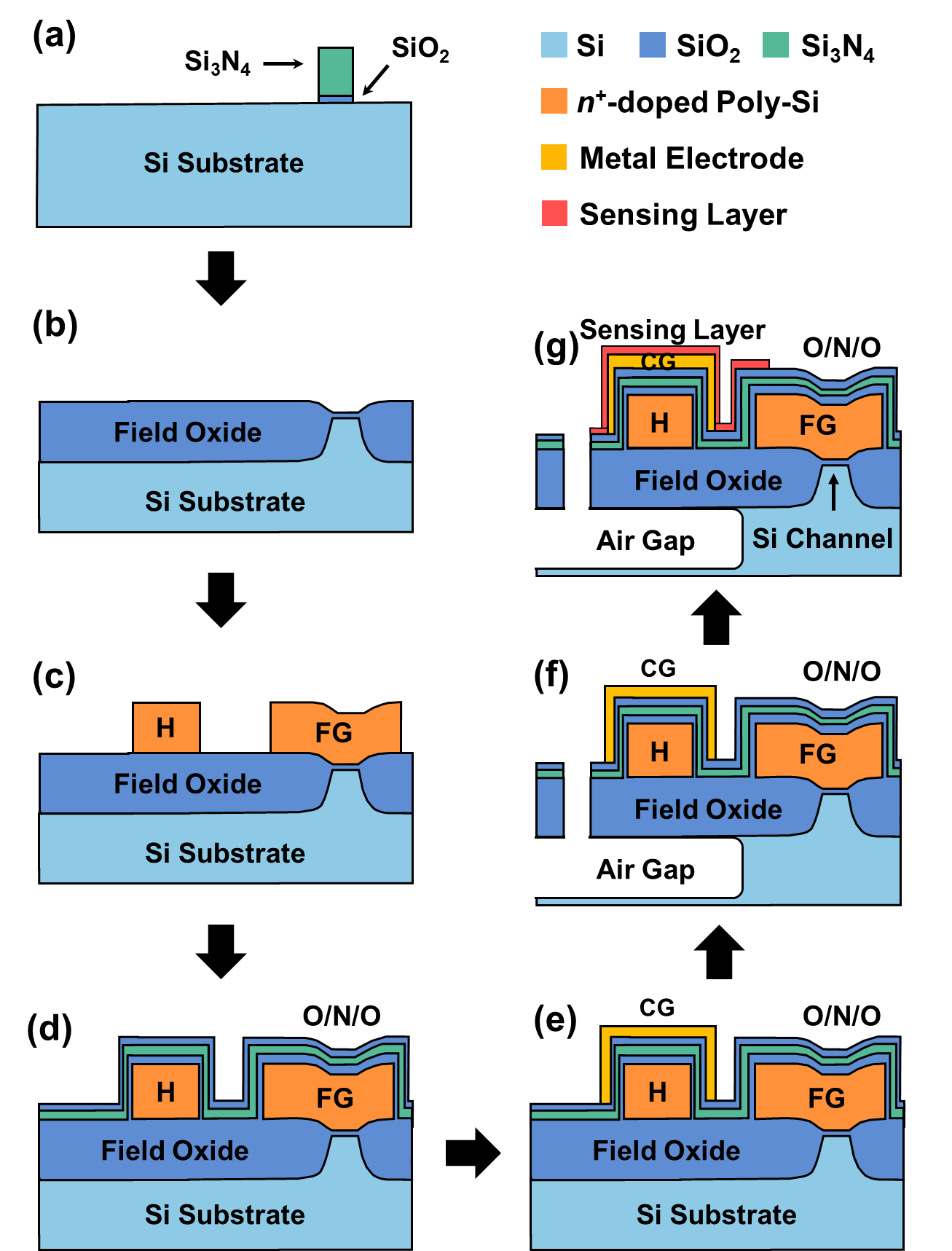


**Figure S11.** Key fabrication process steps of the gas sensor represented by schematic cross-sectional views cut along the channel width direction of the FET transducer.

**Figure S12.** Schematic diagram of the gas measurement system**.**
